# Supplementary material for: Guidance to Best Tools and Practices for Systematic Reviews
Source: JBJS Rev. 2023 Jun 7;11(6):e23.00077. doi: 10.2106/JBJS.RVW.23.00077 (PMC10259219; doi:10.2106/JBJS.RVW.23.00077)
Supplement: Supplementary file 6 [file jbjsrev-11-e23.00077-s006.pdf]

Supplemental file 6: Links to Concise Guide online resources

**Methodological guidance**	
Cochrane	
Intervention	https://training.cochrane.org/handbook/current
Diagnostic	https://training.cochrane.org/diagnostic-test-accuracy-dta-reviews
Prognostic	https://training.cochrane.org/resource/systematic-review-prognosis-studies
Qualitative	https://training.cochrane.org/handbook/current/chapter-21
Overviews	https://training.cochrane.org/handbook/current/chapter-v
JBI	
Intervention	https://jbi-global-wiki.refined.site/space/MANUAL/4688621/Chapter+3%3A+Systematic+reviews+of+effectiveness
Diagnostic test accuracy	https://jbi-global-wiki.refined.site/space/MANUAL/4687355/Chapter+9%3A+Diagnostic+test+accuracy+systematic+reviews
Prevalence and incidence	https://jbi-global-wiki.refined.site/space/MANUAL/4688607/Chapter+5%3A+Systematic+reviews+of+prevalence+and+incidence
Etiology and risk	https://jbi-global-wiki.refined.site/space/MANUAL/4687372/Chapter+7%3A+Systematic+reviews+of+etiology+and+risk
Measurement properties	https://jbi-global-wiki.refined.site/space/MANUAL/4686202/Chapter+12%3A+Systematic+reviews+of+measurement+properties
Umbrella reviews	https://jbi-global-wiki.refined.site/space/MANUAL/4687363/Chapter+10%3A+Umbrella+reviews
Scoping reviews	https://jbi-global-wiki.refined.site/space/MANUAL/4687342/Chapter+11%3A+Scoping+reviews
**Reporting guidelines**	
eMERGe	https://emergeproject.org
ENTREQ	https://doi.org/10.1186/1471-2288-12-181
PRIOR	https://doi.org/10.1136/bmj-2022-070849
PRISMA 2020	http://www.prisma-statement.org/
PRISMA-DTA	http://prisma-statement.org/Extensions/DTA
PRISMA-P	http://www.prisma-statement.org/Extensions/Protocols
PRISMA-ScR	http://www.prisma-statement.org/Extensions/ScopingReviews
SWiM	https://www.bmj.com/content/368/bmj.l6890

**Risk of bias assessment tools for primary studies**	
CASP Qualitative Checklist	https://casp-uk.net/images/checklist/documents/CASP-Qualitative-Studies-Checklist/CASP-Qualitative-Checklist-2018_fillable_form.pdf
JBI Critical Appraisal Checklist for qualitative research	https://jbi-global-wiki.refined.site/space/MANUAL/4687846/Appendix+2.1%3A+JBI+Critical+Appraisal+Checklist+for+Qualitative+Research
Cochrane RoB2	https://methods.cochrane.org/bias/resources/rob-2-revised-cochrane-risk-bias-tool-randomized-trials
COSMIN RoB Checklist	https://www.cosmin.nl/tools/guideline-conducting-systematic-review-outcome-measures.
JBI Critical Appraisal Instrument for Studies Reporting Prevalence Data	https://jbi-global-wiki.refined.site/space/MANUAL/4688355/Appendix+5.1%3A+Critical+Appraisal+Instrument+for+Studies+Reporting+Prevalence+Data
PROBAST	https://www.probast.org/
ROBINS-I	https://methods.cochrane.org/bias/risk-bias-non-randomized-studies-interventions
QUADAS-2	https://www.bristol.ac.uk/population-health-sciences/projects/quadas/quadas-2/
QUIPS	https://www.acpjournals.org/doi/full/10.7326/0003-4819-158-4-201302190-00009
**Overall certainty of evidence**	
For intervention reviews	
GRADE	https://www.gradeworkinggroup.org/
For other review types	
Qualitative	
CERQual	https://www.cerqual.org/
ConQual	https://jbi-global-wiki.refined.site/space/MANUAL/4689627/2.7.3+ConQual+'Summary+of+Findings'
Measurement properties	https://www.cosmin.nl/tools/guideline-conducting-systematic-review-outcome-measures/
**Critical appraisal of systematic reviews**	
AMSTAR-2	https://amstar.ca/Amstar-2.php
ROBIS	http://www.bristol.ac.uk/population-health-sciences/projects/robis/

Kolaski K, Logan LR, Ioannidis JPA. Guidance to best tools and practices for systematic reviews. JBJS Reviews 2023:11(6).
https://doi.org/10.2106/JBJS.RVW.23.00077

Kolaski K, Logan LR, Ioannidis JPA. Guidance to best tools and practices for systematic reviews. JBJS Reviews 2023:11(6).
https://doi.org/10.2106/JBJS.RVW.23.00077
